# Micro-computed tomography analysis of calcium hydroxide delivery efficacy in C-shaped canal system of mandibular second molars

**DOI:** 10.1186/s12903-023-03450-9

**Published:** 2024-01-09

**Authors:** Min Chen, Babita Pradhan, Yajun Meng, Chialing Tsauo, Xuedong Zhou, Dingming Huang, Jingzhi Ma, Yuan Gao

**Affiliations:** 1 State Key Laboratory of Oral Diseases, Department of Cariology and Endodontics, West China Hospital of Stomatology, National Clinical Research Center for Oral Diseases, Sichuan University, 14# 3rd Section, Renmin South Road, Chengdu, Sichuan Province 610041 China; 2 Department of Stomatology, Tongji Hospital, Tongji Medical College, Huazhong University of Science and Technology, No. 1277 Jiefang Avenue, Wuhan, Hubei Province 430030 China; 3 School of Stomatology, Tongji Medical College, Huazhong University of Science and Technology, Wuhan, 430030 China

**Keywords:** Calcium hydroxide, C-shaped canal, Delivery efficacy, Mandibular second molars, Micro-CT

## Abstract

**Background:**

Calcium hydroxide [Ca(OH)_2_] is widely accepted as a biocompatible interappointment intracanal medicament. This study aimed to analyze the efficacy of Ca(OH)_2_ placement into the C-shaped canal system of mandibular second molars using the syringe method with and without lentulo spiral utilizing micro-computed tomography (micro-CT).

**Methods:**

Twenty-four extracted mandibular second molars were instrumented and classified into C-shaped floors (n = 12) and non-C-shaped floors (n = 12). Both groups were placed with Ca(OH)_2_ using the syringe system, then all teeth were scanned and cleaned, and placed with Ca(OH)_2_ again but with the syringe system followed by lentulo spiral and rescanned. The specimens were scanned using micro-CT to analyze the volume, volume percentage, uncontacted surface area, and uncontacted surface area percentage of Ca(OH)_2_ with the two delivery methods in the entire canal and at the apical 4 mm of the canal. Mann-Whitney test and Wilcoxon signed-rank test were used to determine the statistical differences among the groups.

**Results:**

Syringe administration used in conjunction with lentulo spiral presented lower uncontacted surface area, a lower percentage of uncontacted surface area, larger volume, and a higher percentage of volume than syringe without lentulo spiral (P < 0.05). There was no significant difference between the C-shaped floor group and the non-C-shaped floor group (P > 0.05) in the Ca(OH)_2_ uncontacted surface area, volume, and percentages at different regions of canals and among different delivery techniques groups.

**Conclusions:**

The lentulo spiral and syringe technique combination can increase the volume and contacted surface area of Ca(OH)_2_ in the C-shaped canal system of mandibular second molars.

## Background

Elimination of microorganisms and their by-products from an infected root canal system is crucial for successful endodontic treatment. However, it has been reported that some microorganisms may remain lodged in the dentinal tubules even after careful chemo-mechanical preparation [1, 2]. Thus, intracanal medication between sessions can be essential for disinfecting the root canal system, minimizing the risk of reinfection, and favoring periapical tissue repair [3, 4].

Calcium hydroxide is highly recommended and widely accepted as a biocompatible interappointment intracanal medicament [5, 6]. Rajasekharan et al. reported that changes in pH, short-term calcium ion release, and maximum release rate were dependent on the exposed surface area, while maximum calcium ion release was dependent on the volume [7]. Therefore, to maximize the penetration and disinfection properties, Ca(OH)_2_ should ideally be placed deep and in close contact with the canal surface along the working length.

However, the quality of Ca(OH)_2_ placement may depend on canal morphology, instruments, materials, and the delivery technique [8, 9]. Various techniques for intracanal placement of Ca(OH)_2_ have been advocated before [8, 10–15]. It is considered that syringe techniques are easier to use in clinical settings [12], and the lentulo spiral was reported to produce higher filling quality in minimally instrumented canals [10].

The morphology and complexities of the canal system can also influence the effectiveness of intracanal medicament placement [9]. Mandibular second molars with C-shaped canal configurations are especially commonly found in east Asia [16–18]. The main anatomic feature of the C-shaped canal system is the presence of a fin or web connecting the individual root canals, which can harbor many microorganisms even after shaping [19]. Thus, application of intracanal medicament is strongly recommended in these cases.

Traditional methods to verify the quality of Ca(OH)_2_ placement into complex canals are using radiographic images, clearing process and splitting teeth samples [12, 15, 20]. However, these techniques may exhibit subjectivity, lack quantifiability, and possess low resolution. Furthermore, studies using traditional methods reported controversial results of different delivery techniques, including K-type ultrasonic file, Gutta-Condensor, Pastinject, Lentulo spiral, injection system, et al. [13, 21]. Evaluation was done through scoring by examiners [15, 22], calculation of the density of the intracanal medicament and observation of porosities [8, 10, 13, 14, 23], and weight calculation [21]. No previous studies have investigated the percentage of the contacted and uncontacted canal surface area by Ca(OH)_2_, which may more clearly show the contact condition between the intracanal medicament and canal surface.

Micro-computed tomography (micro-CT) has been used in endodontic studies to analyze changes in canal volume, surface area, and uninstrumented surface area after canal preparation [24–26]. To our knowledge, there have been no studies using micro-CT to evaluate the placement efficacy of Ca(OH)_2_. In addition, it is unclear whether lentulo spiral has a significant auxiliary effect on increasing the contact area during placement of intracanal medicament by syringes.

Thus, the purpose of this micro-CT study was to analyze the effectiveness of intracanal placement of Ca(OH)_2_ in C-shaped canals of mandibular second molars using the syringe method with and without lentulo spiral.

## Methods

**Fig. 1:**
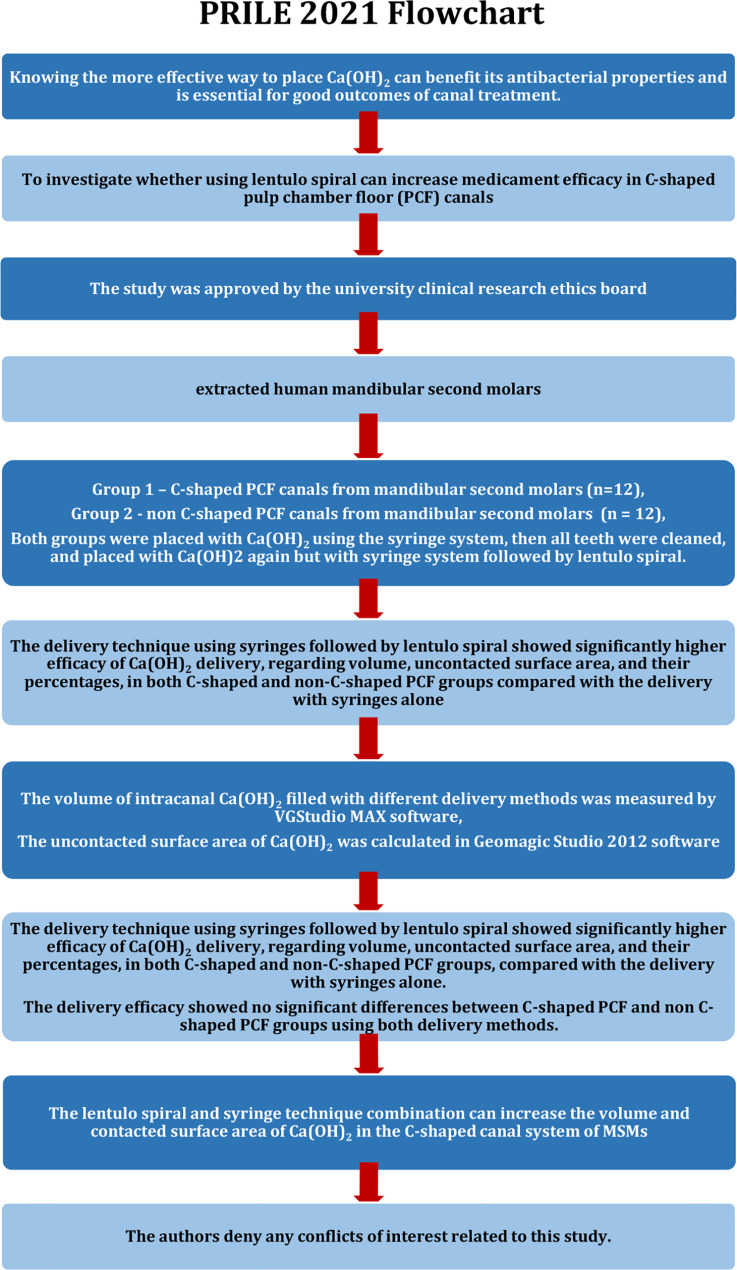
PRILE 2021 flowchart of Ca(OH)_2_ placement efficacy in C-shaped canal system

The manuscript of this laboratory study has been written according to Preferred Reporting Items for Laboratory studies in Endodontology (PRILE) 2021 guidelines (Fig. 1) [27].

### Specimen preparation

80 mandibular second molars from native Chinese patients with fused roots, radicular grooves, and no macroscopically visible external defects were extracted because of severe periodontitis, and were then collected and ultrasonically cleaned. The age and sex were unknown. The study was approved by the university clinical research ethics board (WCHS-IRB-ST-2017-224). The teeth were scanned by micro-CT (µCT-50; Scanco Medical, Brttisellen, Switzerland) at an isotropic voxel size of 30 μm at 90 kVp and 200 mA. All data were exported in DICOM format. The teeth were then three-dimensionally reconstructed using VGStudio MAX 2.0 (Volume Graphics, Heidelberg, Germany) software, and the pulp chamber floor (PCF) was investigated. These teeth were divided into two groups according to a classification based on the shape of the PCF by Min et al. as follows [28]:

C-shaped PCF and orifice: a peninsula-like floor with a continuous or discontinuous C-shaped orifice, which encompasses Types I, II, and III of Min’s classification;

Non-C-shaped PCF and orifice: without peninsula-like floors and with separated mesial and distal canal orifices, similar to Type IV of Min’s classification.

The sample size was calculated based on the data of our pilot study, and the calculation method in a previous study [29]. The analysis was performed using two independent means from the T-­test family in G*Power 3.1 software (Henrick Heine-Universität) with α = 0.05 and 95% power inputs. 10 was considered the minimum sample size to observe significant difference between groups. Considering a low incidence of non-C-shaped PCF canal in fused roots, a final of 24 teeth (12 per group) in the C-shaped PCF and non-C-shaped PCF groups were selected for the study.

Standard access cavities were prepared for all the selected teeth. A size 10 K-type file was introduced into each canal until the tip of the instrument was visualized at the apical foramen when observed under a dental operating microscope (DOM) (Pico; Carl Zeiss, Jena, Germany). Working length (WL) determination was accomplished by subtracting the file 0.5 mm from the total canal length. Root canal preparation was performed using a ProGlider™ Rotary Glide Path File sized 16/0.02 up to Waveone Gold 25/0.07 (Dentsply Maillefer, Ballaigues, Switzerland) mounted on a reciprocating X-Smart Plus motor (Dentsply Maillefer). During mechanical preparation, irrigation was carried out copiously between files with 2 mL of 1% NaOCl. After completion of instrumentation, each canal was irrigated with ultrasonic flush for 30 s using Irrisafe tip (Acteon, Merignac, France), which was inserted to 1 mm short of the WL and activated at 20% power as without cutting power. Each canal was subsequently irrigated with 5 mL 1% NaOCl for 1 min and then dried with paper points.

### Intracanal medicament placement

All roots were embedded with 3 M impression material putty (Express XT, 3 M ESPE, St. Paul, MN, USA) to seal the root structure until the cemento enamel junction (CEJ). UltraCal XS Ca(OH)_2_ paste (Ultradent Products, South Jordan, UT, USA) was applied using the following techniques:

- Group 1: Syringe system with NaviTip needle.
  The 29-G NaviTip needle (Ultradent Products) was attached to the UltraCal syringe and introduced into the canal 2 mm short of the predetermined canal length. The syringe was then gently pressed and withdrawn from the canal until paste reflow was observed and extrusion was evident at the orifices under DOM. Subsequently, a cotton pellet was applied into the canal orifice with Caviton (GC Corporation, Tokyo, Japan) as the temporary filling material. The teeth were stored at 37 °C and 100% humidity for two weeks. Two weeks after the placement of Ca(OH)_2_ paste, the impression material was removed. A second micro-CT scan was carried out for further 3D measurement and analysis.
  Subsequently, the impression material was removed to ensure that the apical foramen was unobstructed, as opposed to being in a sealed state as observed clinically. The Ca(OH)_2_ pastes in all of the teeth were removed using Irrisafe or ET-20 ultrasonic tips powered by a piezoelectric unit (Acteon, Merignac, France). The remaining pastes were further removed using air-scaler-attached EDDY sonic tips (VDW, Munich, Germany), NaOCl, and through a gentle up-and-down motion to ensure free movement of the tips. Then, a final flush was set in an ultrasonic bath (Fisher Scientific Company, Ottawa, Canada). During pilot experiments, these procedures led to nearly complete removal of Ca(OH)_2,_ which was also confirmed by micro-CT inspection; the teeth were then reused in group 2.
- Group 2: Syringe system with NaviTip needle followed by lentulo spiral.
  Compared with group 1, after injection of Ca(OH)_2_ paste, a size 25 lentulo spiral (Dentsply Maillefer) attached to a slow-speed handpiece was inserted 1 mm from working length passively in a clockwise direction at 300 rpm for 10 s. The lentulo spiral was removed, and the UltraCal syringe needle was reinserted into the canal. Ca(OH)_2_ paste was reinjected until paste reflow was observed and extrusion was evident at the orifices under DOM. A cotton pellet was then applied into the canal orifice with Caviton as the temporary filling material. Teeth were stored at 37 °C and 100% humidity for two weeks, and a third micro-CT scan was taken to compare the placement efficacy of different techniques.

### Micro-CT image measurement and analysis

**Fig. 2:**
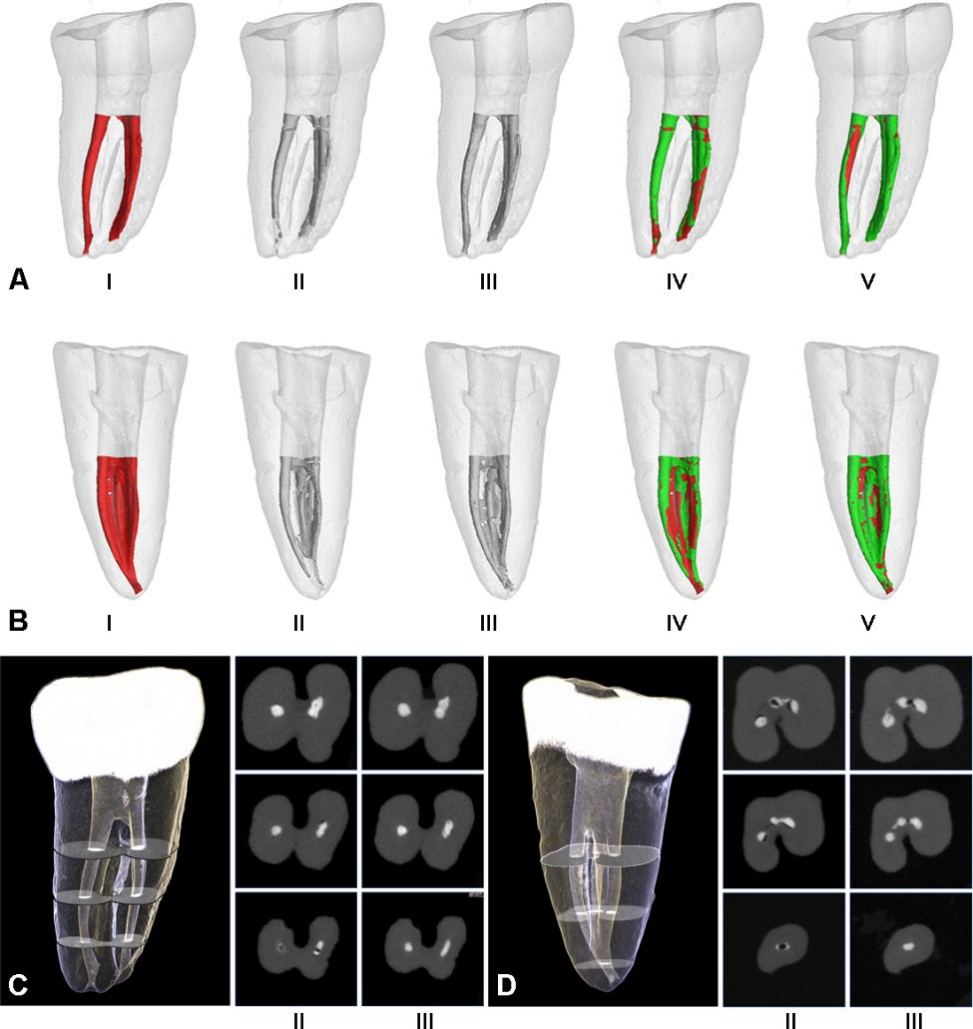
Micro-CT three-dimensional reconstructions of Ca(OH)_2_ placement in C-shaped or non-C-shaped PCF mandibular second molars. (**A-B**) I. Red parts in the models represent the initial post-preparation canal surface area in non-C-shaped PCF and C-shaped PCF, respectively. II-III. grey parts represent Ca(OH)_2_ that was placed into the canal using different delivery techniques. IV-V. green and red parts represent the surface area of the Ca(OH)_2_ that was and was not in contact with the initial canal surface, respectively. II, IV. Ca(OH)_2_ placed with syringes alone. III, V. Ca(OH)_2_ placed with syringes and lentulo spirals. (**C-D**) cross-sections views of the coronal, middle and apical thirds of roots

The measurement methodology is based on a previously published technique by our research team [30]. The DICOM files obtained from the scanned batches were subjected to 3D co-registration using the Elastix rigid image registration module, integrated into the 3D Slicer software (v4.1.1) (Harvard SPL, Boston, MA, USA). Subsequently, the registered data were imported into VGStudio MAX 2.0 for further processing. A semi-automatic threshold-based segmentation technique was employed to create a 3D model of the canal and Ca(OH)_2_, allowing for precise measurements (Fig. 2AI, II, III, 2BI, II, III). The region of interest (ROI) for the Ca(OH)_2_ paste was defined from the canal orifices to 0.5 mm short of the apex (covering the entire canal) and from the canal orifices to the apical 4 mm of the canal.

The volume, uncontacted surface area, and their respective proportions relative to the entire canal and the apical 4 mm region were measured. The VGStudio MAX software was employed for the precise measurement of intracanal Ca(OH)_2_ volume, considering various delivery methods. The determination of the uncontacted surface area of Ca(OH)_2_ was carried out through the following steps: Initially, the region of interest (ROI), comprising both the canal walls and the surface of intracanal Ca(OH)_2_, was digitally reconstructed and exported to 3D STL format using VGStudio MAX software. Subsequently, the surface area of the canal wall that came into contact with Ca(OH)_2_ was computed. This process involved the calculation of the canal surface area and a Boolean intersection using Geomagic Studio 2012 software (Raindrop Geomagic, Morrisville, NC, USA), where each voxel represented 30 μm. In such cases, the Boolean intersection of the canal surface and the contacted surface area of Ca(OH)_2_ equaled zero:

$${\rm{Ca}}{\left( {{\rm{OH}}} \right)_{\rm{2}}}\,{\rm{uncontacted}}\,{\rm{surface}}\,{\rm{area}}\,\left( {\rm{\% }} \right)\,{\rm{ = }}$$ $$=\frac{{\text{C}\text{a}\left(\text{O}\text{H}\right)}_{2}\text{u}\text{n}\text{c}\text{o}\text{n}\text{t}\text{a}\text{c}\text{t}\text{e}\text{d} \text{s}\text{u}\text{r}\text{f}\text{a}\text{c}\text{e} \text{a}\text{r}\text{e}\text{a} \left(\text{i}\text{n} \text{t}\text{h}\text{e} \text{e}\text{n}\text{t}\text{i}\text{r}\text{e} \text{c}\text{a}\text{n}\text{a}\text{l} \text{o}\text{r} \text{a}\text{p}\text{i}\text{c}\text{a}\text{l} 4 \text{m}\text{m}\right)}{ \text{T}\text{o}\text{t}\text{a}\text{l} \text{c}\text{a}\text{n}\text{a}\text{l} \text{s}\text{u}\text{r}\text{f}\text{a}\text{c}\text{e} \text{a}\text{r}\text{e}\text{a} \left(\text{t}\text{h}\text{e} \text{e}\text{n}\text{t}\text{i}\text{r}\text{e} \text{c}\text{a}\text{n}\text{a}\text{l} \text{o}\text{r} \text{a}\text{p}\text{i}\text{c}\text{a}\text{l} 4 \text{m}\text{m}\right)}$$

### Statistical analysis

Mann–Whitney test was used to determine the difference between C-shaped and non-C-shaped groups in the volume and the uncontacted surface area of Ca(OH)_2_ in the entire canal and at the apical 4 mm, and Wilcoxon signed-rank test was used to determine statistical differences between the two different delivery techniques. All statistical tests were performed using SPSS software (SPSS 20.0 for Windows, SPSS, Chicago, IL, USA). A value of p < 0.05 was considered statistically significant.

## Results

**Fig. 3:**
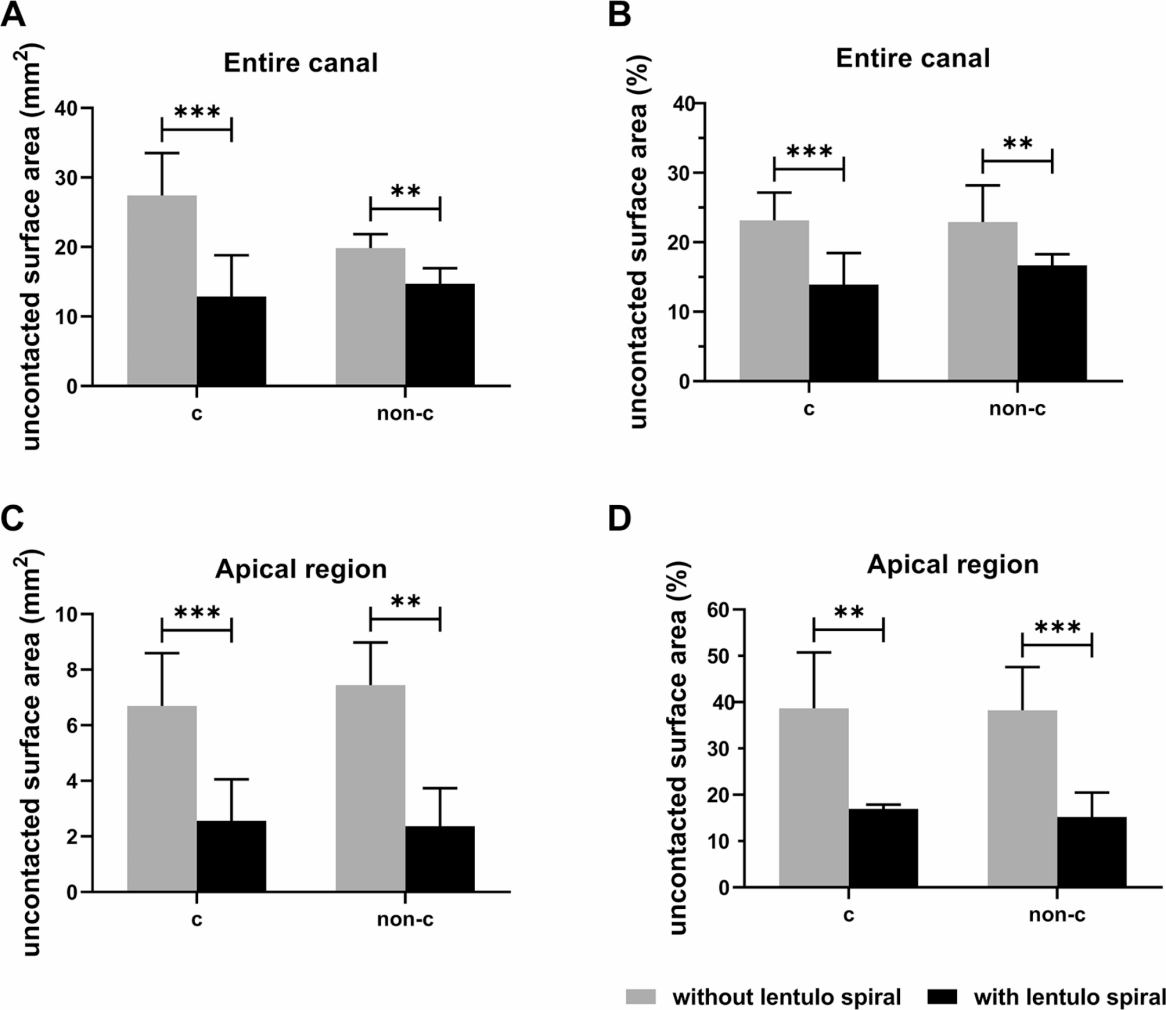
The uncontacted surface area of Ca(OH)_2_ with and without using lentulo spiral. (**A-B**) Uncontacted surface area (mm^2^) and Uncontacted surface area (%) of the entire canal with C-shaped or non-C-shaped PCF. (**C-D**) Uncontacted surface area (mm^2^) and Uncontacted surface area (%) of the apical region with C-shaped or non-C-shaped PCF. *P < 0.05, **P < 0.01, ***P < 0.001

**Fig. 4:**
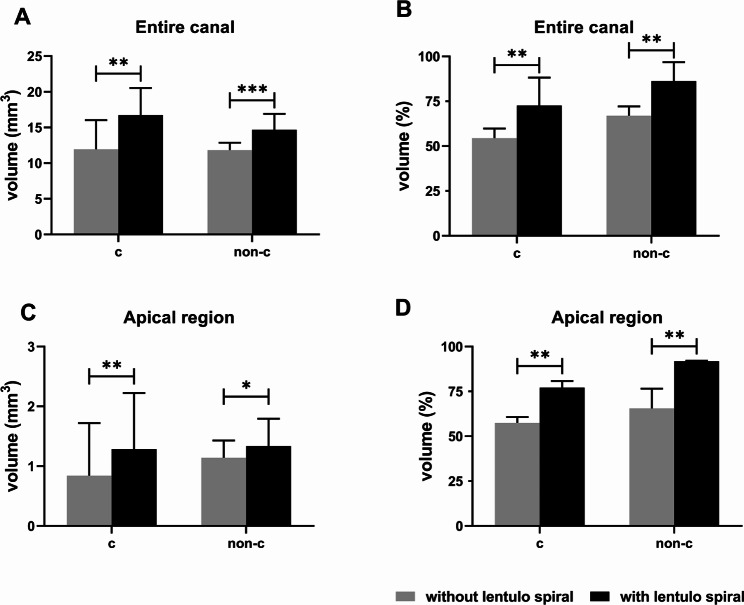
Ca(OH)_2_ volume (mm^3^) and the percentage of volume (%) with and without using lentulo spiral. (**A-B**) Volume (mm^3^) and the percentage of volume (%) of Ca(OH)_2_ in the entire canal with C-shaped or non-C-shaped PCF. (**C-D**) Volume (mm^3^) and the percentage of volume (%) of Ca(OH)_2_ in the apical region with C-shaped or non-C-shaped PCF. *P < 0.05, **P < 0.01, ***P < 0.001

The delivery technique using syringes followed by lentulo spiral showed significantly higher efficacy of Ca(OH)_2_ delivery, regarding volume, uncontacted surface area, and their percentages, in both C-shaped and non-C-shaped PCF groups compared with the delivery with syringes alone (Fig. 2). The median (interquartile range) of the uncontacted surface area of Ca(OH)_2_ after placement with the two techniques in both C-shaped and non-C-shaped PCF groups are shown in Fig. 3. There were statistically significant differences among the two types of delivery techniques in both the C-shaped and non-C-shaped PCF groups (P < 0.001). Specifically, syringe followed with lentulo spiral presented lower uncontacted surface area and a lower percentage of uncontacted surface area than syringe without lentulo spiral, regardless of whether the region of interest encompassed the entire canal (Fig. 3A-B) or the apical 4 mm of the canal (Fig. 3C-D) (P < 0.05).

median (interquartile range) of the volume of Ca(OH)_2_ in the entire canal and apical 4 mm placed with the two techniques in both C-shaped and non-C-shaped PCF groups are shown in Fig. 4. Statistically significant differences were found between the two placement techniques in both C-shaped and non-C-shaped groups, both in the entire canal (Fig. 4A-B) and in the apical 4 mm (Fig. 4C-D) (P < 0.05). Specifically, syringe followed with lentulo spiral showed a larger volume of Ca(OH)_2_ in the entire canal and at the apical 4 mm.

Between the C-shaped and the non-C-shaped PCF groups, there were no statistically significant differences in the mean volume, uncontacted surface area and the percentages for the two delivery techniques, both in the entire canal and at the apical 4 mm (P > 0.05).

## Discussion

Ca(OH)_2_ is the most commonly used intracanal medicament for eliminating residual microorganisms after chemo-mechanical preparation [3, 31]. To obtain the maximum therapeutic benefit of Ca(OH)_2_, the paste must be in direct contact with the root canal walls to allow penetration of the OH^−^ ion and the Ca^2+^ ion [22]. Therefore, in the assessment of Ca(OH)_2_ placement, uncontacted surface area and volume of Ca(OH)_2_ should be essential parameters.

Previous methods used to assess the efficacy of different Ca(OH)_2_ delivery techniques include scoring by an examiner [15, 22], calculation of the density of Ca(OH)_2_ and the porosities [8, 10, 13, 14, 23], and weight calculation [21]. However, scoring by different examiners can result in inaccurate results as it is largely subjective. Simcock et al. reported a strong correlation between the radiographic appearance and the weight after Ca(OH)_2_ delivery into the canal [21]. Calculation of Ca(OH)_2_ density is unable to show the area where the drug is in effect as it does not show where Ca(OH)_2_ is directly in contact with the canal surface. Weight calculation before and after delivery of Ca(OH)_2_ also has a disadvantage that it cannot separately measure the amount of Ca(OH)_2_ in each portion of the canal, for instance, the apical portion. By using the amount of uncontacted surface area as a parameter, the quality of the application of the medicament on the root canal surface can be represented, especially at the apical portion.

For two decades, micro-CT has widely been used in endodontic research. Imaging software allows researchers to calculate and analyze canal surface area before and after instrumentation. No previous study has used micro-CT for assessing the uncontacted or contacted surface area and volume of Ca(OH)_2_ medicament in root canals. In the present study, the surface area of Ca(OH)_2_ was regarded as the pre-preparation area, and the canal surface area was regarded as the post-preparation area. We applied micro-CT scanning to reconstruct a 3D model, which enabled us to calculate and analyze the contacted surface area of the Ca(OH)_2_ in a more standardized and precise manner.

Ca(OH)_2_ paste requires effective delivery into the canal to achieve total antibacterial activity. Several techniques are used for Ca(OH)_2_ placement, among which syringe and lentulo spiral are commonly advocated clinically [23]. Staehle et al. stated that the syringe system provided superior filling quality with Ca(OH)_2_ in straight or lightly curved root canals; the addition of the lentulo spiral was reportedly more effective in filling curved canals [12]. Similar results were also reported in other studies [8, 31, 32].

UltraCal XS Ca(OH)_2_ paste is a commonly used commercial paste with a syringe system. NaviTip syringe ^TM^ is relatively small and can be inserted into the apex of root canals. Thus, two delivery techniques were utilized in the present study, namely NaviTip syringe and NaviTip syringe used in conjunction with lentulo spirals. The results of our study indicated that using lentulo spirals can help to reduce the uncontacted surface area of Ca(OH)_2_ in root canals. This is in agreement with Torre et al.’s study, which compared Ultradent tips, Ultradent tips used in conjunction with lentulo spiral, and lentulo spiral used alone [14]. Their results showed that Ultradent tips used in conjunction with lentulo spiral and lentulo spiral alone were significantly more effective than Ultradent tips alone in the apical 3 mm and 1 mm. Similarly, the syringe-lentulo spiral group in the study by Tan et al. showed a greater mean radiodensity than the syringe-#25 finger spreader group at all levels [23]. As expected in our study, differences in delivery techniques resulted in variations in the uncontacted surface area of Ca(OH)_2_ in both the C-shaped canal systems with C-shaped PCF and non-C-shaped PCF. The group in which NaviTip syringe was used in conjunction with lentulo spiral showed a significantly lower uncontacted surface area. This could be due to the rotation of the lentulo spiral, which may help to remove small, entrapped air bubbles within the canal and spread the Ca(OH)_2_ into the irregular isthmuses and fins in C-shaped canals, thus allowing better distribution and contact of Ca(OH)_2_ with the canal surfaces.

Apart from delivery techniques, the placement quality of Ca(OH)_2_ also depends on the canal morphology. C-shaped canal systems that usually consists of 2 or 3 “main” canals interconnected with fins and isthmuses in the form of a C-configuration in cross section of the root have been reported to have large amounts of debris and uninstrumented areas after preparation [19, 33–35]. Studies have shown that the use of rotary instruments does not improve the situation and no preparation was evident at the interconnecting fins or isthmuses which especially highlights the importance of intracanal medication [36]. Furthermore, the continuously aging of the east Asia population would further complicate this question that not only endodontists but also general dentists may face more and more root canal therapy (RCT) cases related to C-shaped canals owing to extensive caries or abrasion.

Most of the previous studies that focused on intracanal medication delivery techniques were related to the placement of Ca(OH)_2_ into straight and large canals or artificial curved canals(21, 23, 37). No comparable data were reported in C-shaped canals. Therefore, in the present study, mandibular second molars with C-shaped canal system were used to assess the effect of complex canal anatomy on the placement quality of Ca(OH)_2_. Based on our pilot study, most of the Ca(OH)_2_ was removed from the canals after cleaning protocol abovementioned, and the canal space showed no statistical differences to the primary volume, therefore, in the current study, the samples were reused in the lentulo spiral groups to minimize the differences caused by the complex C-shaped canal morphology. There may be potential differences in the pre-medicament baseline in the two techniques, nevertheless, any discrepancies arising from the minute remaining paste were considerably smaller than using an entirely different tooth for comparison. In addition, owing to the C-shaped canal serving as a perfect and well-known ‘complex model’, research methods reported on C-shaped canal systems in this study can also be easily adapted to other studies about other complex human hard tissue anatomy or benchmark quests from the industrial community about related clinical procedures, such as ultrasonic activation/irrigation benchmark test described in this study [38].

The findings of our study indicated that under the investigated conditions, the uncontacted surface area was not influenced by the shape of the PCF in the group filled with the NaviTip syringe system and NaviTip syringe used in conjunction with lentulo spirals. This finding broadly supports the work of Rahde et al., who reported that the curvature did not influence the filling quality of Ca(OH)_2_ into extracted human teeth [20]. However, conflicting results were reported by Sharifi et al., who found that the curvature affected the density of Ca(OH)_2_ [9]. An explanation for this disparity could be the differences in experimental specimens. Sharifi et al. used simulated canals in resin block to assess the effect of the canal curvature while Rahde used extracted human first molars and single-rooted teeth [9, 20].

Contrary to expectations, this study did not find a significant difference in the uncontacted surface area of Ca(OH)_2_ between C-shaped and non-C-shaped PCF groups. Such results may be attributed to the complexity of the root canal systems, as numerous ramifications and isthmuses can exist in C-shaped canal systems with C-shaped and non-C-shaped PCF. Development of novel delivery techniques is needed to improve the application of intracanal medicament in C-shaped canals.

This study showed that an appropriate delivery technique is essential for the clinical application of Ca(OH)_2_ for canal disinfection, especially in complex canal anatomies. The limitation of the study is the small sample size and only one kind of Ca(OH)_2_ pastes, and another limitation is that we used only C-shaped canal system and it may take caution to extrapolate the results to other canals.

## Conclusions

Although complete contact between Ca(OH)_2_ and the canal surface is not yet possible, the combination technique of lentulo spiral and syringe can significantly increase the contacted surface of the root canal wall by Ca(OH)_2_ in C-shaped canal system of mandibular second molars.

## Data Availability

The datasets used and/or analysed during the current study are available from the corresponding author [Jingzhi Ma and Yuan Gao] on reasonable request.
